# Monitoring the maturation of the sarcomere network: a super-resolution microscopy-based approach

**DOI:** 10.1007/s00018-022-04196-3

**Published:** 2022-02-23

**Authors:** Anna Skorska, Lisa Johann, Oleksandra Chabanovska, Praveen Vasudevan, Sophie Kussauer, Maximilian Hillemanns, Markus Wolfien, Anika Jonitz-Heincke, Olaf Wolkenhauer, Rainer Bader, Hermann Lang, Robert David, Heiko Lemcke

**Affiliations:** 1 Department of Cardiac Surgery, Reference and Translation Center for Cardiac Stem Cell Therapy (RTC), Rostock University Medical Center, 18057 Rostock, Germany; 2 Faculty of Interdisciplinary Research, Department Life, Light and Matter, University Rostock, 18059 Rostock, Germany; 3 Department of Systems Biology and Bioinformatics, University of Rostock, Rostock, Germany; 4 Research Laboratory for Biomechanics and Implant Technology, Department of Orthopedics, Rostock University Medical Center, 18057 Rostock, Germany; 5 Department of Operative Dentistry and Periodontology, Rostock University Medical Centre, 18057 Rostock, Germany; 6 Stellenbosch Institute of Advanced Study (STIAS), Wallenberg Research Centre at Stellenbosch University, Stellenbosch, 7602 South Africa

**Keywords:** iPSC cardiomyocytes, Sarcomere network, Maturation, Super resolution microscopy

## Abstract

The in vitro generation of human cardiomyocytes derived from induced pluripotent stem cells (iPSC) is of great importance for cardiac disease modeling, drug-testing applications and for regenerative medicine. Despite the development of various cultivation strategies, a sufficiently high degree of maturation is still a decisive limiting factor for the successful application of these cardiac cells. The maturation process includes, among others, the proper formation of sarcomere structures, mediating the contraction of cardiomyocytes. To precisely monitor the maturation of the contractile machinery, we have established an imaging-based strategy that allows quantitative evaluation of important parameters, defining the quality of the sarcomere network. iPSC-derived cardiomyocytes were subjected to different culture conditions to improve sarcomere formation, including prolonged cultivation time and micro patterned surfaces. Fluorescent images of α-actinin were acquired using super-resolution microscopy. Subsequently, we determined cell morphology, sarcomere density, filament alignment, z-Disc thickness and sarcomere length of iPSC-derived cardiomyocytes. Cells from adult and neonatal heart tissue served as control. Our image analysis revealed a profound effect on sarcomere content and filament orientation when iPSC-derived cardiomyocytes were cultured on structured, line-shaped surfaces. Similarly, prolonged cultivation time had a beneficial effect on the structural maturation, leading to a more adult-like phenotype. Automatic evaluation of the sarcomere filaments by machine learning validated our data. Moreover, we successfully transferred this approach to skeletal muscle cells, showing an improved sarcomere formation cells over different differentiation periods. Overall, our image-based workflow can be used as a straight-forward tool to quantitatively estimate the structural maturation of contractile cells. As such, it can support the establishment of novel differentiation protocols to enhance sarcomere formation and maturity.

**Supplementary Information:**

The online version contains supplementary material available at 10.1007/s00018-022-04196-3.

## Introduction

The generation of induced pluripotent stem cell (iPSCs) was a hallmark in stem cell research, enabling the generation of almost any cell type from individual donors. Since their derivation does not require the use of human embryos ethical problems are avoided [1, 2]. Nowadays, cardiomyocytes (CM) derived from iPSCs are commonly used for human disease modeling, therapeutic applications and drug-testing approaches [3, 4]. The latter on has been successfully demonstrated by the CiPA initiative, which was initiated to assess the proarrhythmic risk of novel cardio-therapeutics [5].

Great progress has been made during the last decade to establish, control and improve the differentiation of CMs from iPSCs. However, the immature phenotype of these CMs is a major limitation for their pre-clinical and clinical use [6, 7]. This cardiogenic maturation involves multiple developmental changes of the cellular physiology, including morphological alterations, electrophysiological changes, metabolic maturation, and establishment of a proper contraction machinery [7]. The development of a sufficient contraction capacity strongly depends on the sarcomere content and proper organization of the myofilaments. In adult CMs, the sarcomere length is ~ 2 µm and the respective filaments are aligned perpendicular to the longitudinal axis to generate maximum contraction forces. In contrast, iPSC-CMs often demonstrate a reduced sarcomere length and disorganized filaments, scattered throughout the cytoplasm [8]. Furthermore, the formation of a proper contraction network encompasses changes in gene expression and isoform switching of sarcomeric proteins. For example, while fetal CMs preferably express slow skeletal cardiac troponin I (*TNNI1*), cardiac troponin I (*TNNI3*) is mainly detected in mature CMs [6, 9]. Similarly, splicing modifications have been found for titin and the regulatory light chain of myosin [6, 10].

Hence, a variety of protocols have been applied to push iPSC-CMs towards a more mature phenotype. The cultivation in a 3D microtissue environment, mechanical stimulation, editing of contraction-related genes or optimized medium, containing oxidative substrates, have been found to increase the maturation of the sarcomere network and promote cell contraction [11–14]. The success of these protocols is tightly associated with precise monitoring of the contraction apparatus, which implies evaluation of parameters describing the degree of sarcomere maturation. Using appropriate techniques, it is possible to control and improve the differentiation conditions needed to promote the establishment of a functional sarcomere network.

Several previous studies have analyzed some aspects of sarcomere organization, such as orientation and sarcomere length, using conventional confocal images and different software tools [15–18]. We have applied some of these techniques to develop a strategy for the standardized evaluation of the sarcomere network of contractile cells, including iPSC-derived CMs and skeletal muscle cells, by acquisition of multiple parameters defining the maturation status of the contraction machinery. We compared the structural organization of iPSC-CMs, subjected to maturation-favored culture conditions, with CMs isolated from neonatal and adult tissue. To quantitatively assess the quality of the sarcomere network we applied super-resolution microscopy and deep machine learning. These techniques allow the collection of important data on sarcomere maturation, including myofibril density and orientation, sarcomere length and z-Disc thickness of the filaments. Using this approach, we showed that sarcomere maturation can be profoundly improved when iPSCs-CMs are gown on structured surfaces that facilitate the development of adult-like cell morphology. Similarly, long-term culture had beneficial effects on sarcomere organization and density. Likewise, we successfully monitored the sarcomere maturation of skeletal muscle cells, depending on the differentiation time. Based on the results we suggest our strategy to be suitable for the evaluation of sarcomere maturation and, thus, highly valuable to establish new protocols aiming to improve structural maturity of in vitro generated contractile cells.

## Material and methods

### Cell culture and differentiation conditions

iPSCs (Takara Bio Inc, Kusatsu, Japan) were cultured in iPS Brew (Miltenyi Biotec, Bergisch Gladbach, Germany), supplemented with Zellshield (Biochrom, Berlin, Germany). The general procedure of cardiac differentiation was adopted from the protocol published previously [15]. Briefly, to induce cardiac differentiation, cells were seeded on Laminin521 (BioLamina, Sundbyberg, Sweden) coated surfaces and cultured in RPMI 1640 Glutamax media (Thermo Fisher, Waltham, MA, USA) containing 1% sodium pyruvate, 200 µM ascorbic acid (all Sigma Aldrich, St. Louis, USA) 1% Zellshield and 2% B27 without insulin (Miltenyi Biotec) and treated with 1 µM Chir99021, 5 ng/mL basic fibroblast growth factor, 5 ng/mL bone morphogenetic protein 4 and 9 ng/mL Activin A (all Miltenyi Biotec) for 3 days. Afterwards, cells were grown in RPMI 1640 Glutamax supplemented with B27 with insulin (Miltenyi Biotec) and 5 mM IWP-2 (Tocris, Bristol, UK) for 7 days to ensure cardiogenic differentiation. Finally, metabolic selection was performed using RPMI 1640 without Glucose (Thermo Fisher), containing 1% Zellshield, 2.3 mM sodium acetate and 100 µM mercaptoethanol (all Sigma Aldrich).

After 25 and 40 days, differentiated iPSC-CMs were dissociated using a dissociation kit (Stemcell Technologies, Vancouver, Canada) according to the manufacturer’s instructions and seeded on glass coverslips and chamber slides for immune labeling and microscopic analysis. To investigate the impact of surface topography, cells were seeded on structured surfaces, containing trench-like depressions with 20 µm width (4D cell, Montreuil, France).

CM maturation was further improved by treatment with Thyroid hormone (R&D systems, Minneapolis, USA) and dexamethasone (Sigma). Following 25 days of differentiation, CMs were cultivated in media containing 100 nM Thyroid hormone and 1 µM dexamethasone for 7 days, while medium was renewed daily. Control cells were treated with vehicle (DMSO).

Human skeletal muscle cells were purchased from Pro Vitro (Berlin, Germany) and cultivated at low passage in skeletal muscle cell growth medium as recommended by the manufacturer (ProVitro). 5 × 10^4^ myoblasts were seeded onto collagen I (rat tail)- coated glass coverslips (NeuVitro, Vancouver, WA, USA) and incubated for 1, 3 and 4 days in differentiation medium (ProVitro), without medium exchange. Afterwards, cells were fixed with 2% paraformaldehyde, followed by immune labeling.

### Isolation of murine adult and neonatal CMs

All studies involving adult and neonatal mice were performed according to the ethical guidelines for animal care of the Rostock University Medical Centre.

Isolation and culture of adult murine CMs was conducted as described elsewhere [19]. Upon injection of collagenase 2/4 solution (Sigma Aldrich) into the left ventricle, heart tissue was cut into small pieces and single cells were obtained by trituration with a 1 mL pipette. Isolated CMs were seeded on collagen-coated surfaces and subjected to fluorescence labeling.

Isolation of neonatal CMs was performed as described previously [20]. Briefly, following enzymatic digestion, isolated cells were seeded on gelatin-coated 8 well chamber slides (Ibidi) or glass coverslips and cultured in DMEM supplemented with 10% FBS (Pan Biotech, Aidenbach, Germany) and 1% Zellshield (Biochrom).

### Labeling of the sarcomere network

Initially, CMs were fixed with 2% paraformaldehyde for 15 min, followed by incubation with 0.2% Triton-X 100 (all Sigma Aldrich) for 5 min. Upon treatment with 1% bovine serum albumin, the sarcomere network of CMs and skeletal muscle cells was visualized by labeling with anti-sarcomeric α-actinin (abcam, ab9465, dilution 1:100) and goat anti-mouse AlexaFlour647 secondary antibody (Thermo Fisher, A-21237, dilution 1:200).

### Super resolution microscopy

For structured illumination microscopy (SIM) image acquisition, labeled cells on glass coverslips were mounted with Fluoroshield™, containing DAPI (Thermo Fisher). Images were recorded with the 40 × alpha 1.46 Plan apochromat objective with oil immersion (Zeiss, Oberkochen, Germany). Z-stacks were recorded in SIM mode with a 16 bit depth at 5 angles, with averaging 4; 51 µm grid was applied for 633 laser line. The acquired SIM dataset were reconstructed by the ZEN software (Zeiss).

To perform PALM imaging, labeled cells were kept in an imaging solution containing 10% Glucose, 10 mM sodium chloride, 50 mM Tris–HCl, catalase, Pyranose oxidase, 100 mM Cysteamine, 2 mM Cyclooctatetraene and 100 mM Mercaptoethanol (all Sigma Aldrich). The detailed process of image acquisition was reported previously [21]. A total of 5000–10,000 frames were acquired using a Zeiss ELYRA LSM 780 imaging system (Zeiss) equipped with a 1.57 N.A. 100 × oil objective. Subsequently, reconstruction of raw images and post-processing was performed with Image J software and the Thunderstorm plugin [22].

### Image analysis

Image analysis and evaluation of all parameters was conducted with Image J software. For evaluation of cell morphology, cell borders were marked manually, followed by the acquisition of aspect ratio, circularity and roundness. Sarcomere network density was determined by generation of binary images using Image J ridge detection plugin [23]. The filament density was calculated as percentage from the overall cell area.

For detection of sarcomere orientation, microscopic images were aligned according to the longitudinal axis of the cell and orientation was quantified using directionality and orientation plugin for Image J [24–26].

Sarcomere length was determined by measuring the distance of two intensity peaks of neighboring filaments. For each cell, the length of 20 sarcomeres was detected. For Z-Disc thickness, the width of 50 filaments per cell was automatically calculated with the Image J integrated ridge detection plugin as described previously.

### Calcium imaging

Calcium imaging was performed using Calbryte520 calcium dye (AAT Bioquest, Inc., Sunnyvale, USA). Cells were loaded with 5 µM dye diluted in a mixture of culture medium and PBS (1:1). After 30 min cells were washed with pre-warmed medium. Videos were acquired with Zeiss ELYRA LSM 780 imaging system at 83fps. Assessment of calcium kinetics was done by the CalTrack tool [27].

### RNA isolation and quantitative real-time polymerase chain reaction

RNA isolation of cardiomyocytes was carried out using the NucleoSpin^®^ RNA isolation kit (Macherey-Nagel, Düren, Germany) according to the manufacturer instructions. Subsequent cDNA synthesis was performed with the High-Capacity cDNA Reverse Transcription Kit (Thermo Fisher Scientific).

For qRT-PCR analysis, samples were loaded on a StepOnePlus™ Real-Time PCR System (Applied Biosystems, Foster City, USA) with following reaction parameters: start at 50 °C for 2 min, initial denaturation at 95 °C for 10 min, denaturation at 95 °C for 15 s and annealing/elongation at 60 °C for 1 min with 45 cycles. For one PCR reaction, 30 ng cDNA were used and mixed with the following Taqman^®^ target gene assays: ACTN2 (Hs00153809_m1); MYH6 (Hs01101425_m1), TNNI3 (Hs00165957_m1), GAPDH (Hs02786624_g1) (all Thermo Fisher). CT values were normalized to GAPDH and data were calculated as fold-change expression, related to control.

### Machine learning

The 2D projections of the SIM images were acquired with Image J via a maximum illumination projection along the z-axis. Subsequently, images were analyzed using the AMES Network. The AMES network is a Convolutional Neural Network trained to rate 2D images of CMs based on their sarcomere structures [28]. Similar to the manually conducted image analysis, AMES rates CMs with a dense, well-organized sarcomere network higher than ones with loosely organized filaments.

### Statistical analysis

All data are presented as mean ± standard error of the mean (SEM). Graphs represent data from three independent experiments, while *n* is the total number of cells included into analysis. Cells were obtained from 2 to 4 different cardiac differentiation experiments. Statistical significance was calculated using a one-way or two-way ANOVA, followed by Bonferroni or Dunnett’s post hoc tests for multiple comparisons. For comparison of two groups, Students *t* test was applied. Probability levels considered as statistically significant were *p ≤ 0.05, **p ≤ 0.01, ***p ≤ 0.001 and ****p ≤ 0.0001. Calculations and graph analysis were performed using GraphPadPrism5 software (GraphPad Prism, Inc., San Diego, CA, USA).

## Results

### Topographical cues promote an adult-like morphology of iPSC-CM

**Fig. 1:**
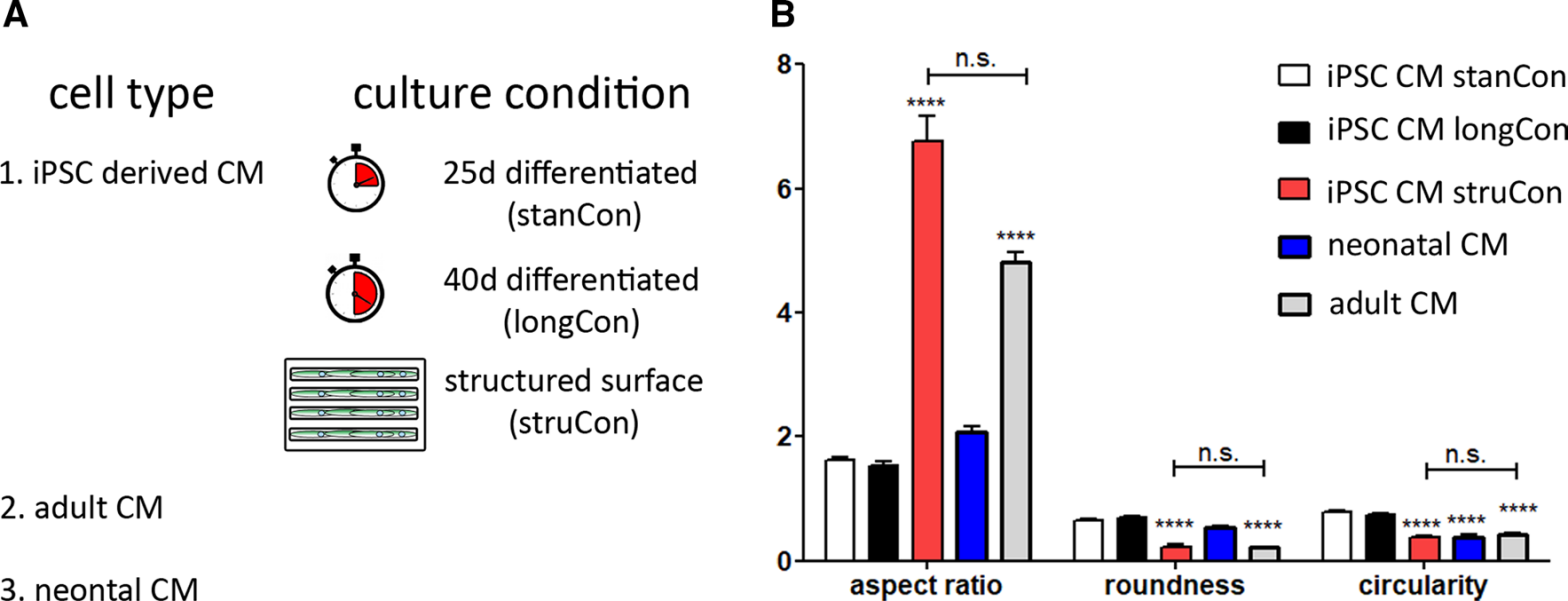
Morphological parameters of iPSCs CMs subjected to different culture conditions. **A** Sarcomere maturation was analyzed in cardiac and skeletal muscle cells, subjected to different culture conditions. iPSC CMs were either differentiated for (I) 25 days (stanCon), (II) 40 days (longCon), or (III) seeded on structured surfaces (struCon). CMs obtained from adult and neonatal tissue were used as reference cells. **B** While iPSC-CMs, grown on plan surfaces (stanCon, longCon), share high similarity with neonatal CMs, cultivation on structured surfaces (struCon) promotes elaboration of an adult-like cell shape, indicated by increased aspect ratio, reduced roundness and circularity. Statistical analysis of shape descriptors was performed using two-way ANOVA, followed by Bonferroni post hoc test, *n* = 50, ***p < 0.001

To optimize the structural maturation of iPSC-derived CMs we used three different culture conditions. (I) Cells were differentiated and cultured for 25 days (iPSC CM stanCon), while in a (II) second treatment cells were differentiated for 40 days (iPSC CM longCon). Additionally, (III) 25 day differentiated CMs were cultivated on micropatterned surfaces, containing lines of 20 µm width (iPSC CM struCon). For validation of our approach and for proper evaluation of the maturation state, iPSC-derived CMs were compared with CMs isolated from neonatal and adult heart tissue (Fig. 1A). The latter was assumed to represent the optimal level of morphology and sarcomere maturation, while neonatal cells were considered as a pre-mature state.

Initially, we determined cellular morphology since cell shape is closely linked to the degree of sarcomere organization. We defined several shape descriptors of all cell types, including aspect ratio, roundness and circularity (Fig. 1B). Primary CMs from adult tissue are rod shaped with an aspect ratio of ~ 5 and low values for roundness and circularity, indicating smooth cell borders. In contrast neonatal cells as well as iPSC-derived CMs, differentiated for 25 and 40 days, demonstrated a round and irregular morphology, leading to a low aspect ratio and significantly increased roundness and circularity values. However, cultivation of iPSCs-CMs on micropatterned surfaces promotes a cellular shape that resembles the morphology of adult CMs, shown by similar shape parameters (Fig. 1B).

### Evaluation of sarcomere density

Next, we asked whether the different culture conditions influence the structural maturation of the actinin network. Compared to standard confocal imaging, our super-resolution microscopy-based approach enables more accurate data acquisition and analysis as shown in Fig. S1 and in our previous studies [21].

**Fig. 2:**
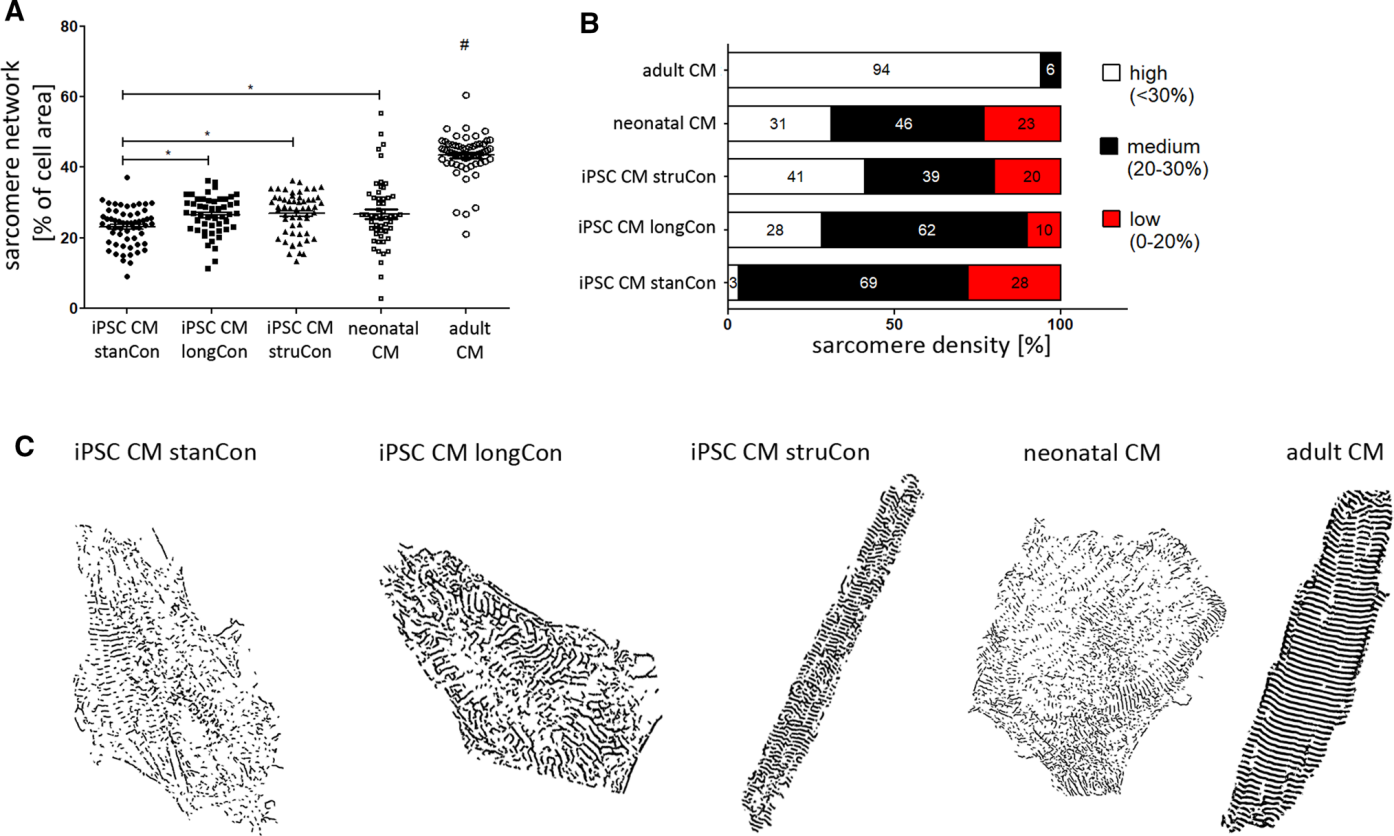
Density of the sarcomere network in iPSC CMs subjected to different culture conditions. **A** The mean sarcomere density of iPSC CMs was comparable to neonatal cells and significantly increased upon cultivation under optimized conditions (iPSC CM longCon, iPSC CM struCon). **B** A profound increased number of cells with high sarcomere density was detected after prolonged cultivation time (iPSC CM longCon). This effect was even more pronounced when cells were grown on structured surfaces (iPSC CM struCon). **C** Binary images of the α-actinin network indicate the difference in sarcomere density upon treated groups. Note that cell size was modified for better visualization of sarcomere filaments. Statistical analysis of sarcomere density was performed using one-way ANOVA, followed by Dunett’s post hoc test, *n* = 41–50, *p < 0.05, **p < 0.01, ***p < 0.001, ^#^*p* < 0.001 compared to all groups

**Fig. 3:**
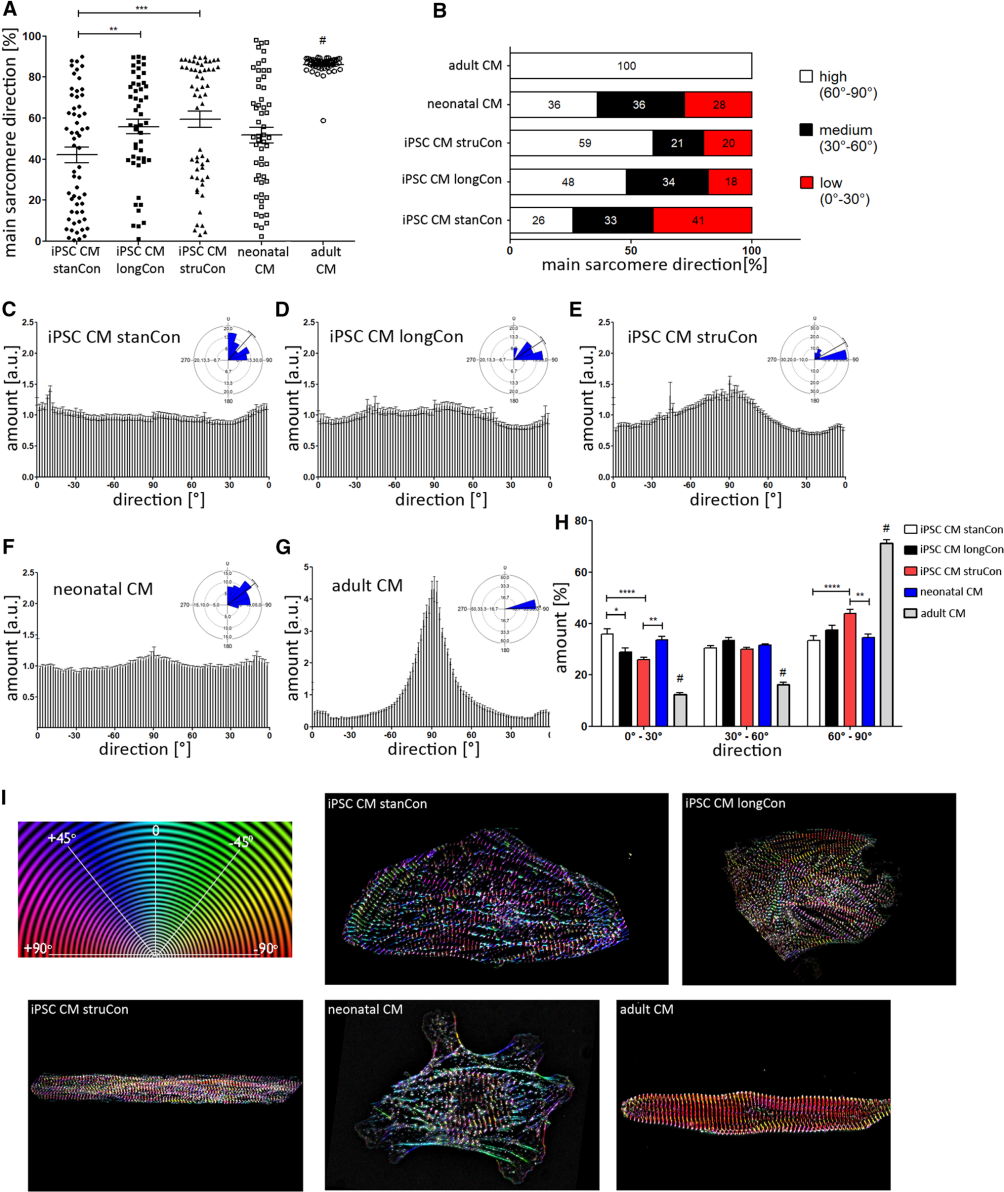
Analysis of sarcomere orientation upon optimized culture conditions. **A** The main direction of sarcomere filaments in iPSC CMs was similar as detected in neonatal cells. However, cultivation on structured surfaces (iPSC CM struCon) significantly improved the orientation of the sarcomere network, if compared to standard culture conditions (iPSC CM stanCon). **B** Contingency plot shows that optimized culture conditions increased the number of cells containing filaments with high direction quality (60°–90°), while the portion of cells with low directionality decreased. **C**–**G** Consistently, histograms and rose diagrams confirm the beneficial effects of prolonged culture period and structured surfaces on filament orientation, shown by peak formation between − 60° and 60°. **H** Quantitative assessment of filaments at given direction intervals confirm the increase of structures with higher orientation. **I** Representative images of treated groups with color-coded directionality of sarcomere structures (inserted color wheel). A high degree of orientation is found in adult CM (red color), while large amounts of green and blue color represent low sarcomere orientation, shown in neonatal CMs and iPSC CMs, cultured under standard conditions. Statistical analysis of sarcomere directionality was performed using Watson–Williams test, *n* = 41–58, *p < 0.05, **p < 0.01, ***p < 0.001, ^#^*p* < 0.001 compared to all groups

At first, we investigated the overall density of the sarcomere network (“sarcomerization”), i.e. the percentage of sarcomere filaments per cell area. As expected, adult CMs demonstrated by far the highest amounts of α-actinin structures. Quantitative analysis revealed that adult CMs showed a ~ 40% increased content of sarcomeres per cell, when compared to iPSC-derived CMs (Fig. 2A). However, if compared to standard culture conditions, the prolonged culture of iPSC CMs and the application of structured surfaces resulted in an elevated sarcomere density that was comparable to neonatal cells (iPSC CM stanCon vs. iPSC CM longCon vs. iPSC CM struCon vs. neonatal CM: 23.13 ± 0.71 vs. 26.46 ± 0.76 vs. 26.91 ± 0.81 vs. 26.77 ± 1.31, Fig. 3A).

Further, cells were classified into three categories, according to their sarcomere amount (low: 0–20%, medium: 20–30% and high: < 30%). The contingency plot in Fig. 2B, clearly visualizes a profound shift from medium and low quality cells to high level network density in iPSCs that were subjected to optimized culture conditions. Binary images of α-actinin labeled cells, shown in Fig. 2C, confirmed the difference of sarcomere density in treated groups.

### Analysis of sarcomere orientation

A high amount of α-actinin fibers along with proper alignment of the sarcomere filaments is necessary to ensure maximum force generation and optimal cell contraction. Hence, we analyzed the overall orientation of the α-actinin network related to the longitudinal axis of the cell. In adult CMs, the main direction was 86.08° ± 0.69°, indicating that most structures are aligned almost perpendicular to the cell axis, which corresponds to an optimal established contraction network. In contrast, iPSC-derived CMs exhibited a significantly reduced main orientation that, however, could be drastically improved by optimized culture conditions (Fig. 3A). Upon cultivation of iPSC CMs on micropatterned surfaces, the main direction of the sarcomere network increased by ~ 40% (iPSC CM stanCon vs. iPSC CM struCon: 42.07 ± 3.08 vs. 59.45 ± 3.89). A significant beneficial effect on filament orientation was further observed in cells that have been subjected to an extended cultivation period (iPSC CM stanCon vs. iPSC CM longCon: 42.07 ± 3.08 vs. 55.81 ± 3.51). Following application of these optimized culture conditions, the number of iPSC-derived CMs exhibiting high filament orientation (60°–90°) rises by ~ 2-fold, while the number of cells with a low main direction (0°–30°) declines in the same manner when compared to standard conditions (Fig. 3B).

These findings are supported when having a closer look on the distribution of individual filament populations. Figure 3C–E shows histograms of all tested cell types giving the amount of filaments related to the respective orientation range. Fully mature CMs from adult tissue are characterized by a pronounced peak between a direction of − 80°/+ 80° (Fig. 3G). On the contrary, iPSC-derived CMs, differentiated for 25 days, demonstrated no visible peak formation, indicating the disorganized sarcomere phenotype (Fig. 3C). Similar to the overall direction (Fig. 3A), an improvement of cell culture parameters led to an increased amount of filaments in the direction range of − 80°/+ 80° (Fig. 3D, E). This effect was even more distinct in iPSC CMs grown on micro structured slides. Quantitative analysis of three different direction intervals (0°–30° vs. 30°–60° vs. 60°–90°) confirmed the positive influence of culture duration and surface patterning on sarcomere maturation. The amount of structures in the lower direction range (0°–30°) decreased, while more filaments became oriented at the optimal orientation (60°–90°) (Fig. 3H).

These differences in sarcomere alignment are illustrated in representative SIM images (Fig. 3I) that visualize filament orientation in a color-coded manner. The more colors are present in the SIM image, the higher the level of disorganized α-actinin structures. While adult CMs are mainly composed of a single color, images from neonatal cells and iPSC CMs contain a broad range of coloring. However, long-term cultured iPSC CMs and the use of patterned surface exhibited a less colorful α-actinin network in comparison to neonatal cells and iPSC CMs differentiated under standard conditions.

**Fig. 4:**
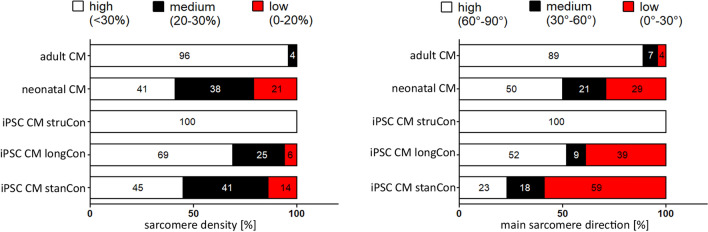
Analysis of sarcomere density and direction by machine learning. Automatic machine learning was applied to evaluate sarcomere density and orientation. According to the algorithm-based classification, iPSC CM, grown on structured surfaces demonstrate the highest maturation level, comparable to adult CMs. Prolonged culture conditions also result in an increased number of cells with improved sarcomere density and orientation, if compared to the standard culture protocol. *n* = 12–20

To prove the quality of our manual based evaluation, we additionally applied deep machine learning, estimating the level of network density and filament orientation. For that purpose, SIM images were rated into three categories (low, medium, high) that correspond to the state of sarcomere maturation. Consistent with our manual based quantification adult CMs and iPSC-CMs grown on micro structured surfaces demonstrated the highest maturation level regarding network density and sarcomere alignment (Fig. 4). Moreover, the algorithm revealed that a prolonged cultivation period improves the establishment of proper sarcomere filaments.

### Architecture of the sarcomere network

**Fig. 5:**
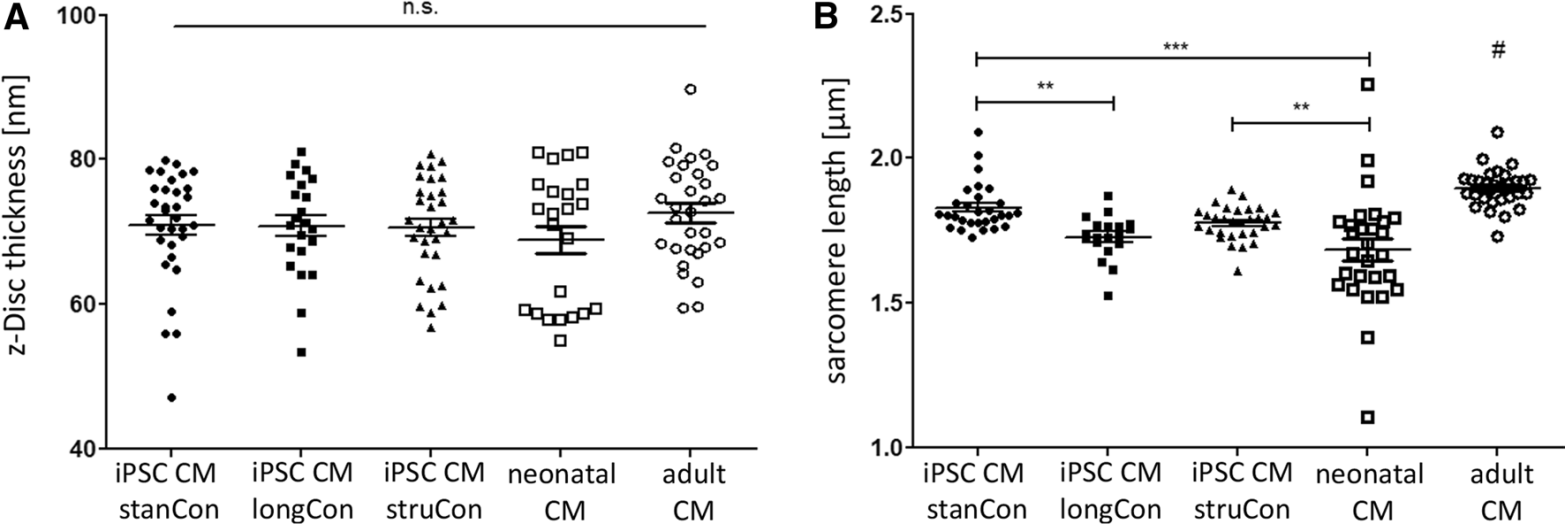
Analysis of z-Disc thickness and sarcomere length. **A** Evaluation of z-disc thickness did not show any differences between iPSC CMs, adult, and neonatal cells. **B** Adult CMs demonstrated the longest sarcomeres among all tested groups. Optimized culture conditions for iPSC CMs were found to have no profound benefits regarding sarcomere length. Statistical analysis was performed using one-way ANOVA, followed by Dunett’s post hoc test, *n* = 20–33, **p < 0.01, ***p < 0.001, ^#^*p* < 0.05 compared to all groups

Following analysis of sarcomere density and orientation, we intended to monitor the architecture of the α-actinin network, including z-Disc thickness and sarcomere length. Using the PALM technique, we evaluated the thickness of individual actinin filaments and found no significant differences between iPSC-CMs, neonatal as well as adult CMs (Fig. 5A). However, measurement of the sarcomere length revealed distinct variations among tested groups (Fig. 5B). With a size of 1.90 ± 0.01 µm, adult CMs demonstrated the longest sarcomeres. The sarcomere length of iPSC CM was significantly reduced, ranging from 1.73 ± 0.02 to 1.82 ± 0.02. Interestingly, a prolonged culture period resulted in a lower sarcomere length, when compared to control iPSCs. The cultivation on micro patterned surfaces had no beneficial effect on the molecular dimensions of the sarcomere network.

### Sarcomere maturation following thyroid hormone stimulation

**Fig. 6:**
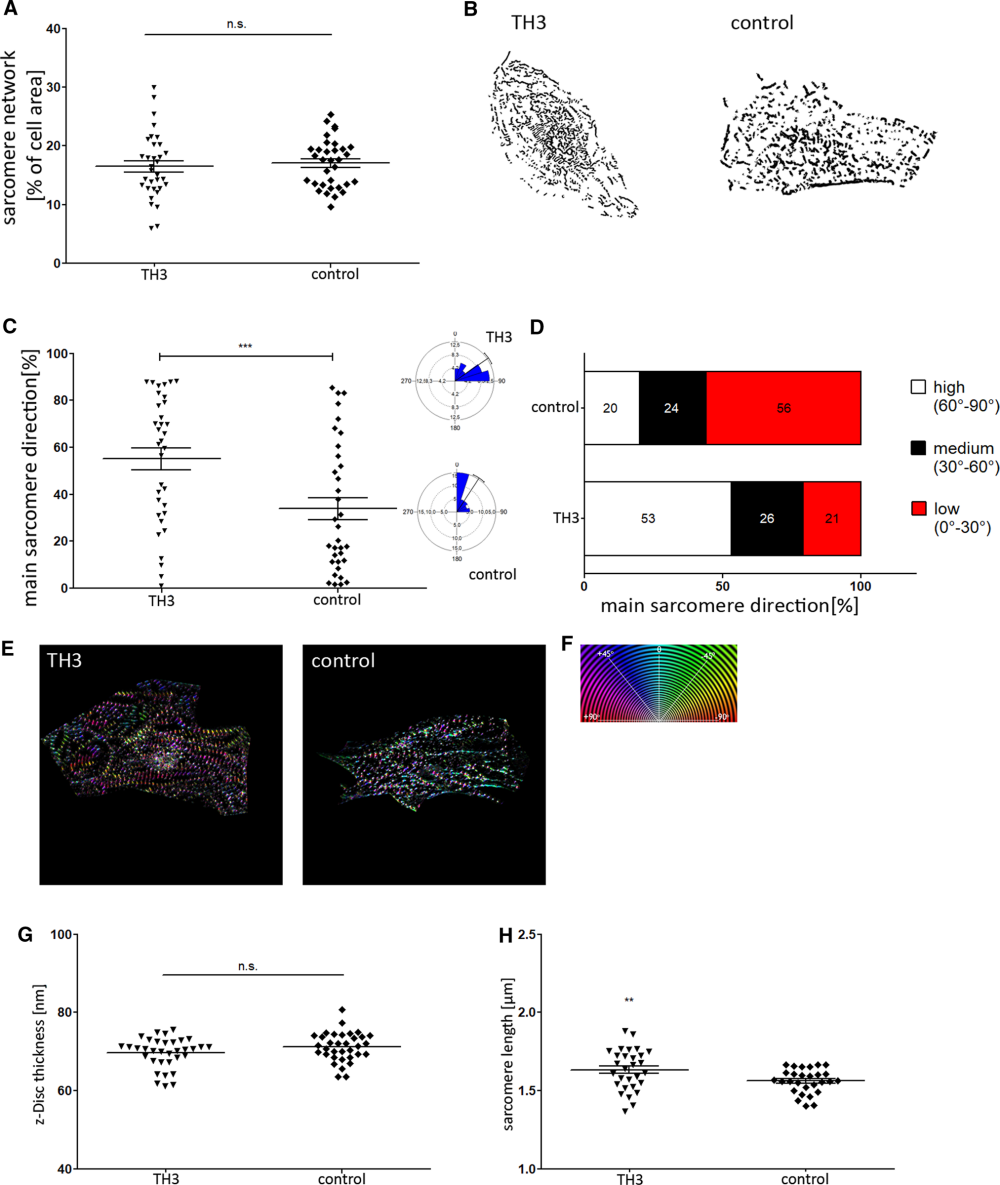
Effect of thyroid hormone and dexamethasone (TH3) on sarcomere network of iPSC CMs. **A**, **B** Application of TH3 did not result in an increase of sarcomere content. **C**, **D** However, main direction of filaments was improved following TH3 incubation as well as the proportion of cells with high filament orientation. **E** Representative images of TH3 and control group with color-coded directionality of sarcomere structures. **G** Z-disc thickness was not influenced by TH3 stimulation. **H** In contrast, length of sarcomeres was found to be significantly increased compared to control cells. Statistical analysis was performed using Students *t* test and Watson–Williams Test for filament directionality, *n* = 30, **p < 0.01, ***p < 0.001

In another approach we applied thyroid hormone (TH3) and dexamethasone, previously shown to improve CM maturation, to further test our evaluation strategy [29, 30]. Following our image analysis, we found that the sarcomere density was not affected by TH3 (Fig. 6A,B).

However, compared to control cells, the filament orientation (TH3 vs. control: 55.09 ± 3.73 vs. 33.84 ± 4.73) as well as the sarcomere length (TH3 vs. control: 1.63 ± 0.024 vs. 1.56 ± 0.014) were significantly enhanced following TH3 incubation (Fig. 6C,H), confirming the suitability of our approach to evaluate sarcomere maturation.

### Calcium handling properties of iPSC CMs

**Fig. 7:**
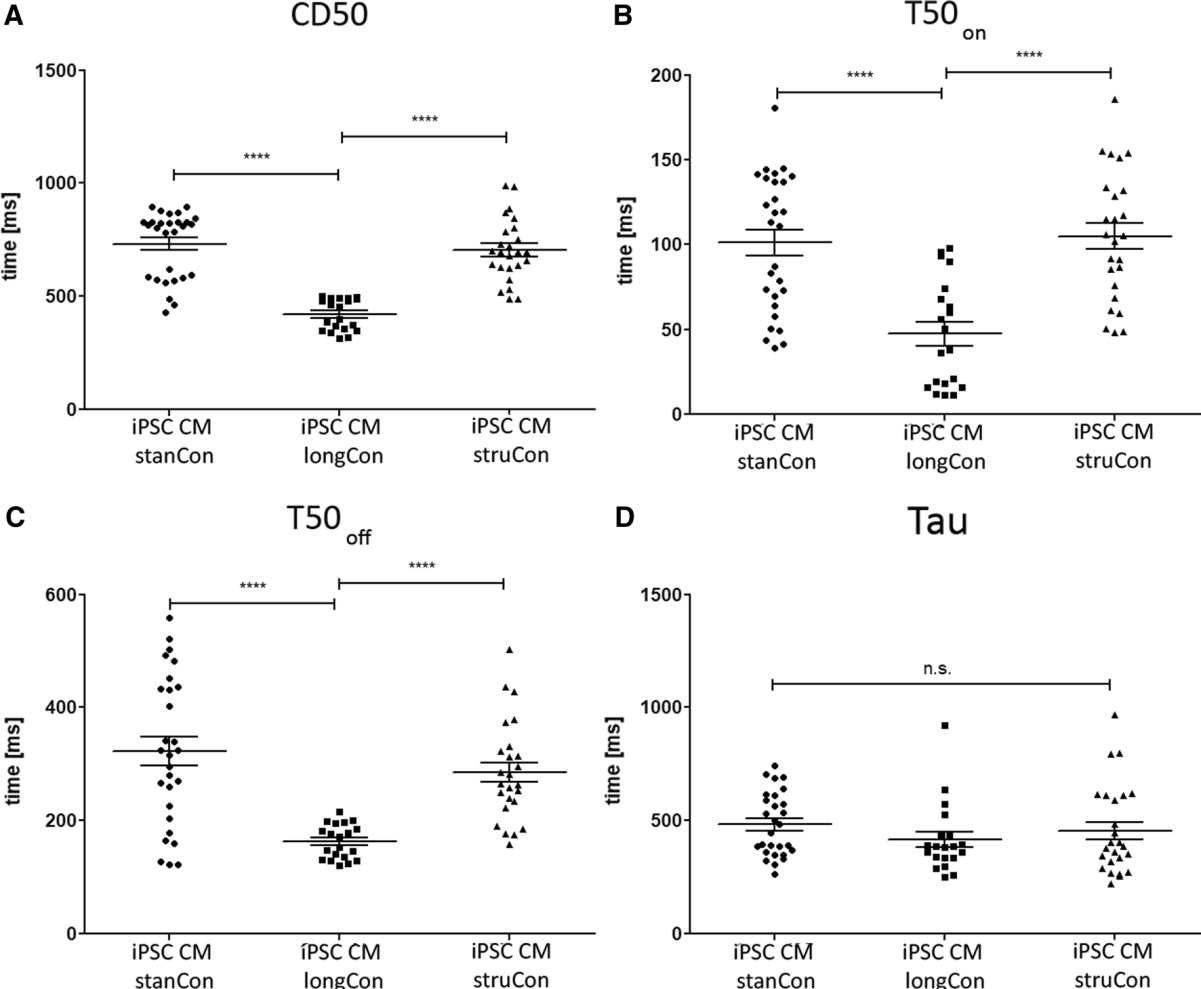
Calcium kinetics of iPSC CMs at optimized culture conditions. **A** The time when the calcium signal is 50% above the baseline (CD50) was profoundly lower in cells cultured for longer periods. **B**, **C** Likewise, upstroke (T50_on_) and downstroke (T50_off_) time were reduced under these conditions. **D **Similarly, calculated calcium decay (tau) was found to be slightly decreased when cells where subjected to longer culture time. While a prolonged CM cultivation induce the strongest effect, structured surfaces also improve calcium handling, yet without statistical significance. Statistical analysis was performed using one-way ANOVA, followed by Dunett’s post hoc test, *n* = 20–28, ***p < 0.001

Further, we asked whether the differences in maturation, observed for the sarcomere network, are reflected in a varying calcium handling ability. Therefore, cells were loaded with a calcium sensitive dye and several calcium kinetic parameters were assessed. As shown in Fig. 7, optimized culture conditions improve calcium handling, while prolonged CM culture was found to induce the strongest effect (Fig. 7A–D). The time when the calcium signal is 50% above the baseline (CD50) is profoundly lower in cells cultured for 40 days (iPSC CM stanCon vs. iPSC CM longCon vs. iPSC CM struCon: 731.9 ± 27.94 vs. 420.2 ± 15.64 vs. 704.8 ± 27.68, Fig. 7A). Likewise, a prolonged culture period results in a significantly reduced upstroke (T50_on_, iPSC CM stanCon vs. iPSC CM longCon vs. iPSC CM struCon: 101.1 ± 7.60 vs. 47.45 ± 7.05 vs. 104.8 ± 7.63, Fig. 7B) and downstroke time (T50_off_, iPSC CM stanCon vs. iPSC CM longCon vs. iPSC CM struCon: 322.6 ± 24.85 vs. 162.9 ± 6.75 vs. 285.3.8 ± 17.51, Fig. 7C). The calcium decay (Tau) was also decreases, though no statistical significance was detected (iPSC CM stanCon vs. iPSC CM longCon vs. iPSC CM struCon: 482.8 ± 26.43 vs. 415.2 ± 34.49 vs. 455.5 ± 39.22, Fig. 7D). Taken together, these data show that sarcomere maturation of iPSC CMs is accompanied by improved calcium handling properties.

### Sarcomere maturation in skeletal muscle cells

**Fig. 8:**
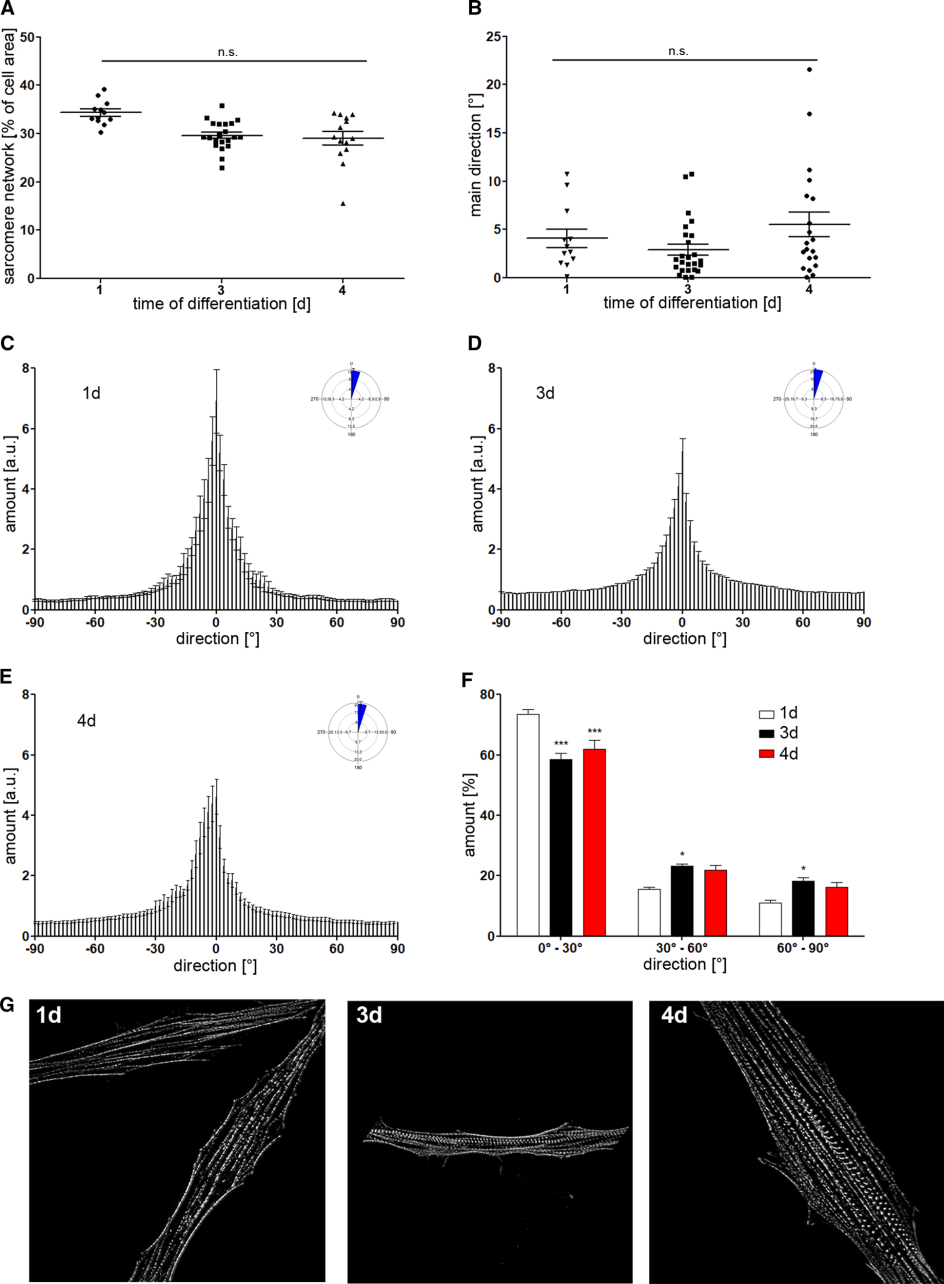
Evaluation of sarcomere maturation in human skeletal muscle cells. **A**, **B** Density of the sarcomere network and main orientation was monitored during differentiation of human skeletal muscle cells (day 1, 3 and 4). **C**–**E** Histograms rose diagrams of sarcomere orientation demonstrate the distribution of filaments. Most sarcomere structures were found to align parallel to the longitudinal cell axis. **F** Prolonged differentiation leads to a reduced amount of structures between 0°–30°, while the number of sarcomere filaments with improved orientation (30°–90°) was increased. **G** Representative images of α-actinin labeled skeletal muscle cells at different timepoints of differentiation. Statistical analysis was performed using Watson–Williams Test for filament orientation and one-way and two-way ANOVA, followed by Dunett’s post hoc test, *n* = 12–21, *p < 0.05, ***p < 0.001 compared to 1d

We showed that our image-based approach is suitable to quantitatively evaluate the sarcomere maturation in iPSC-derived CMs. To test a broader applicability of our approach, we transferred it to a non-cardiac cell type and analyzed the sarcomere network of human skeletal muscle progenitors. The level of sarcomere maturation was modified by varying the period of differentiation to 1, 3 and 4 days, followed by labeling of the sarcomere filaments and subsequent image-based evaluation of network density and orientation. Representative SIM images of the α-actinin network are given in Fig. 8G.

One day after induction of differentiation, skeletal muscle cells were found to contain the highest, though not significant, amount of filaments, if compared to prolonged differentiation time (Fig. 8A) The overall orientation was detected to be similar between all treated groups (Fig. 8B). However, the corresponding histograms demonstrated a slight change of certain filament populations, indicating a shift from lower to higher directionality, depending on the differentiation conditions (Fig. 8C–E). Indeed, quantitative analysis confirmed the differences observed in the histograms. The number of sarcomere structures between 0° and 30° was significantly reduced in cells that have been differentiated for 3 and 4 days. Concomitantly, the amount of filaments oriented between 30° and 90° was increased, indicating a more mature sarcomere phenotype (Fig. 8F).

Based on these data, we conclude that our approach is not only restricted to cardiac cells but can also be applied to any contractile cell type that contains a sarcomere network.

## Discussion

The generation of CMs resembling an adult-like phenotype is one of the main objectives in cardiovascular research. Although large progress has been made in the development of iPSC-derived CMs, limited maturation is still a major obstacle that impedes their use in basic research and for clinical applications. The proper maturation of cardiac cells encompasses the establishment of a functional contraction machinery. Most studies targeting the structural maturity of iPSC-derived CMs focus only on sarcomere length and cell morphology, thus, lacking information regarding sarcomere alignment and integrity [14, 31, 32]. Hence, we have defined a set of parameters, acquired by super-resolution microscopy, to precisely evaluate the quality of the sarcomere network in contractile cells, including CMs and skeletal muscle cells. Application of culture conditions stimulating structural maturity showed the suitability and reliability of this technique to monitor sarcomere formation, alignment and integrity. As this imaging-based strategy considers the structural aspects of sarcomere maturation, contractility data were not assessed. In this regard, traction force microscopy or the use of fluorescent micropillars can help to correlate sarcomere quality and contraction force, complementing our microscopic approach [33–36]. In addition, the formation of a mature sarcomere network encompasses isoform switching of myofibril proteins, including titin and myosin heavy chain protein, which should also be considered to fully address sarcomere maturation [7].

In adult heart tissue, rod-shaped CMs are aligned longitudinally to ensure optimal heart contraction. In contrast, iPSC-derived CMs are tending to demonstrate a circular morphology when cultured on plane surfaces [22, 37], which is confirmed by our morphological analysis (Fig. 1). This morphological and structural phenotype of iPSC CMs is more comparable to immature, neonatal cardiac cells that also exhibit an irregular morphology and a disorganized contraction apparatus [8, 22, 38]. A fact that is verified by our approach, showing that CMs derived from iPSCs share more similarities with neonatal CMs as with native cardiac cells (Figs. 2, 3, 5).

However, there a plenty of strategies to facilitate the structural maturation of in vitro generated cardiac cells. The application of patterned surfaces with trench-like depressions allowed iPSC-CMs to obtain a rod-shaped phenotype that is comparable to native CMs regarding aspect ratio, circularity and roundness parameter. Using our microscopic approach, we have quantitatively determined a clear benefit of structured surfaces for sarcomere density and, even more profound, on sarcomere orientation (Figs. 2, 3). Likewise, several previous studies have identified a pro-maturing effect of topographical cues on sarcomere orientation, indicating the tight relationship between cell morphology and elaboration of an optimal contraction machinery [39–42]. These micro patterned surfaces provide topographical signals that support spreading and elongation of the cell as well as the organization of the cytoskeletal network, leading to more aligned sarcomere filaments. Ribeiro and colleagues demonstrated that the use of substrates providing physiological shape improve the alignment of sarcomere filaments [43]. Likewise substrate stiffness was shown to influence sarcomere organization and contraction properties [44]. Although we did not assess the mechanical properties of iPSC-CMs, one could also expect an improved contraction capacity. Former studies already detected a strong correlation between the degree of sarcomere orientation and cell contractility, independent on the technique used to support the structural maturation [32, 37, 45, 46]. To detect differences in filament orientation we subjected our datasets to circular statistics. Due to its periodic nature, circular data requires specific analysis methods that are different from linear statistical tests [47].

The whole maturation process of CMs in vivo takes several years and it was discovered that long term in vitro culture similarly promote the elaboration of a more mature structural phenotype, including the formation of Z-, A-, H- and M-bands, improved myofibril density and sarcomere organization [48–50]. In accordance to these results, we determined an elevated filament density and a more aligned arrangement of sarcomere structures in cells subjected to prolonged cultivation time (Figs. 2, 3). Notably, this effect of both long term culture and surface patterning on sarcomere orientation was even more evident when individual filament populations were analyzed. In addition, we applied hormonal cues, which was previously shown to influence sarcomere formation [29]. In accordance with these data we found a positive effect of Thyroid hormone and dexamethasone on sarcomere organization using our evaluation approach (Fig. 6). Moreover, improvement of sarcomere maturation correlates with better calcium handling properties (Fig. 7), which is in line with former studies, showing that the application of pro-maturation stimuli leads to faster calcium kinetics of iPSC CMs [29, 51, 52].

Beside cardiac cells, the presented approach was further applied to skeletal muscle cells. Here, the differentiation from progenitor cells leads to formation of a less profound sarcomere network if compared to iPSC-derived CMs (Fig. 6). Nevertheless, our approach enabled us to detect changes in sarcomere alignment with increased differentiation time. The detection of these subtle structural alterations relies on the applied SIM technique that allows image acquisition with higher resolution. Conventional fluorescence imaging does not provide sufficient structural details, instead, leads to more blurry microscopic images (Fig. S1) [33, 53, 54]. However, the visualization of individual filaments or filament bundles is crucial to obtain reliable data regarding sarcomere organization.

One limitation of our study is the comparison of human iPSC-derived CMs and murine cells. However, since isolated adult and neonatal CMs from human tissue are difficult to obtain, the use of primary murine cells as reference marker is a common procedure for comparative studies of CM structure and function [55–58]. In both, human and murine CMs sarcomere filaments are aligned perpendicular to the longitudinal axis for optimal force generation. Hence, the presented directionality data are not affected by the interspecies comparison. For the sarcomere length, previous studies have reported distances between adjacent z-discs of 1.75–2.3 µm, depending on the sample and the techniques applied for image acquisition [59–62]. Similarly, we determined a sarcomere length of ~ 2 µm in adult murine CMs, thus highly reflecting the mature phenotype of human CMs. Therefore, we conclude that murine cells represent an appropriate reference cell type to evaluate the structural maturation of iPSC CMs, although one cannot exclude slight differences in sarcomere architecture among different species.

Taken together, our approach enables the quantitative assessment of alterations of the sarcomere network in contractile cells. Using this technique, we demonstrated that structural maturation can be facilitated by specific culture conditions. In particular, patterned surfaces had a profound effect on sarcomere orientation in iPSC-derived CMs. Beside this optimization of cardiac and skeletal muscle cell differentiation protocols, our method can also be valuable tool in disease modeling as some cardiac dysfunctions and myopathies have been shown to be related to an impaired sarcomere integrity [63–67].

## Supplementary Information

Below is the link to the electronic supplementary material.

Figure S1 Comparison of SIM and standard confocal imaging on same cardiomyocyte population. (A) SIM enabled higher lateral resolution, visualizing sarcomere filaments with increased accuracy compared to confocal imaging. (B) Hence, the individual filaments are thinner which results in a decreased sarcomere content. (C, D) Moreover, the main filament direction differs between both microscopy techniques, showing increased directionality in SIM images (TIF 11296 KB)

Figure S2 Gene expression of sarcomeric proteins in iPSC CMs subjected to optimized culture conditions. Compared to cells cultured for 25 days, prolonged cultivation and the application of structured surfaces was found to increase the gene expression sarcomeric proteins (TIF 695 KB)

## Data Availability

Datasets that have been generated in the current study are available upon reasonable request and on the Zenodo platform (10.5281/zenodo.5862000).

## Code availability

Applied plugins for image analysis are available via respective references.
