# Supplementary figures and images for: Monitoring the maturation of the sarcomere network: a super-resolution microscopy-based approach
Source: Cell Mol Life Sci. 2022 Feb 23;79(3):149. doi: 10.1007/s00018-022-04196-3 (PMC8866374; doi:10.1007/s00018-022-04196-3)

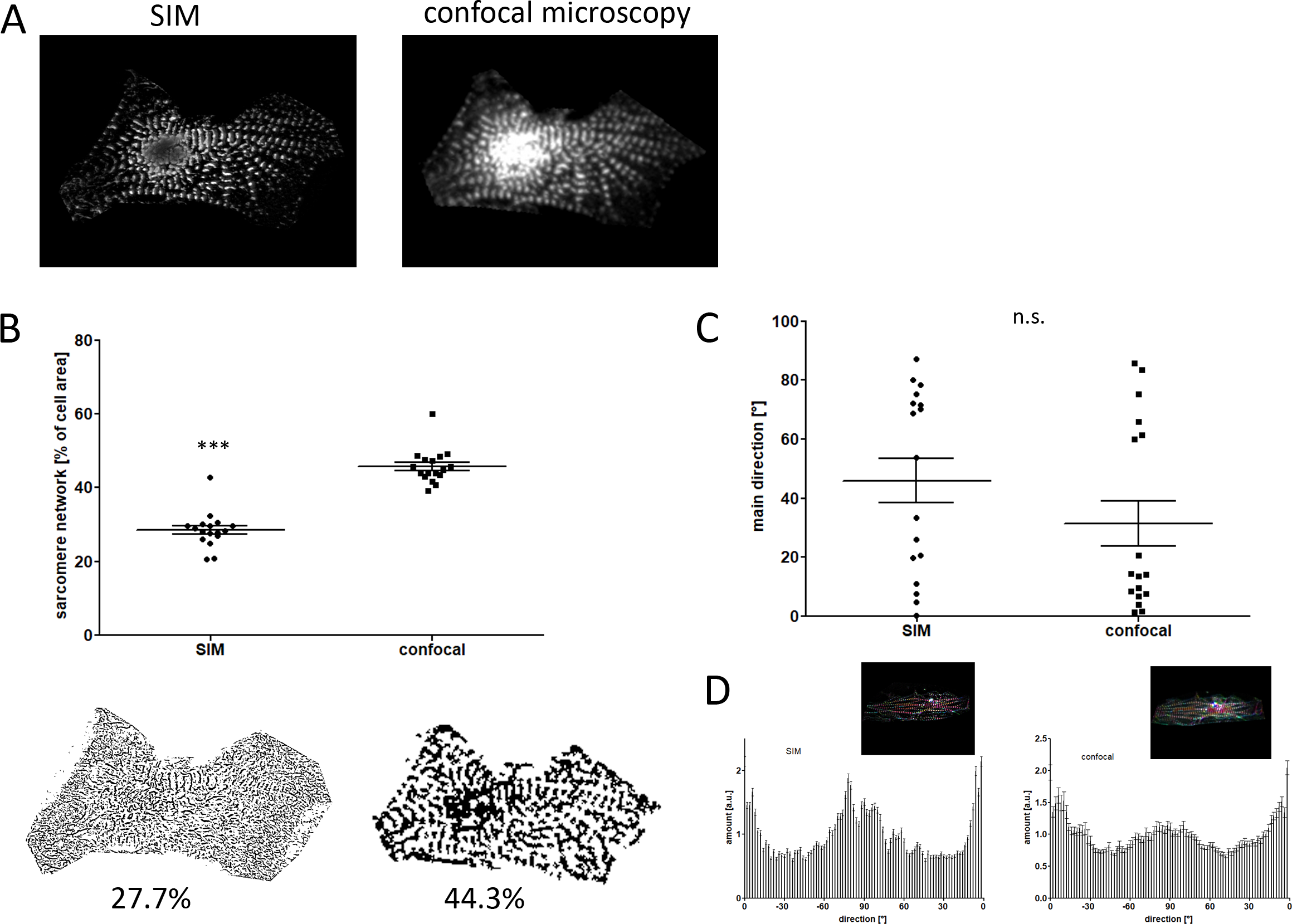

Supplement: Supplementary file 1 — Figure S1 Comparison of SIM and standard confocal imaging on same cardiomyocyte population. (A) SIM enabled higher lateral resolution, visualizing sarcomere filaments with increased accuracy compared to confocal imaging. (B) Hence, the individual filaments are thinner which results in a decreased sarcomere content. (C, D) Moreover, the main filament direction differs between both microscopy techniques, showing increased directionality in SIM images (TIF 11296 KB) [file 18_2022_4196_MOESM1_ESM.tif]

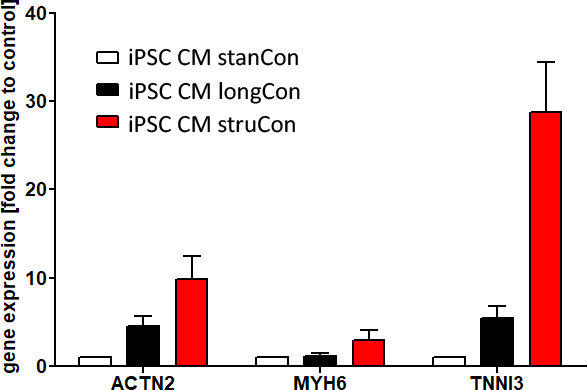

Supplement: Supplementary file 2 — Figure S2 Gene expression of sarcomeric proteins in iPSC CMs subjected to optimized culture conditions. Compared to cells cultured for 25 days, prolonged cultivation and the application of structured surfaces was found to increase the gene expression sarcomeric proteins (TIF 695 KB) [file 18_2022_4196_MOESM2_ESM.tif]
